# Dietary vitamin D and calcium and periodontitis: A population-based study

**DOI:** 10.3389/fnut.2022.1016763

**Published:** 2022-12-22

**Authors:** Gustavo G. Nascimento, Fábio R. M. Leite, David A. Gonzalez-Chica, Karen G. Peres, Marco A. Peres

**Affiliations:** ^1^Section for Periodontology, Department of Dentistry and Oral Health, Aarhus University, Aarhus, Denmark; ^2^National Dental Research Institute Singapore, National Dental Centre Singapore, Singapore, Singapore; ^3^Oral Health ACP, Duke-NUS Medical School, Singapore, Singapore; ^4^Discipline of General Practice, Faculty of Health and Medical Sciences, Adelaide Medical School, The University of Adelaide, Adelaide, SA, Australia; ^5^Oral Health ACP, Health Services and Systems Research Programme, Duke-NUS Medical School, Singapore, Singapore

**Keywords:** periodontal disease, diet, nutrition, micronutrients, epidemiology

## Abstract

**Aim:**

This study aimed to explore the relationship between dietary vitamin D and calcium intake and periodontitis among adults and whether it differs from males to females.

**Methods:**

Cross-sectional analysis of a population-based cohort study with adults aged 20 to 60 from Southern Brazil. Intake of vitamin D and calcium were gathered in 2012 using two 24h-dietary recalls. Clinical examination assessed the clinical attachment level and bleeding on probing. Confounders included sex, age, family income, smoking, and obesity. The controlled direct effect of vitamin D and calcium on periodontitis was examined using marginal structural modeling. Analyses were also stratified by sex.

**Results:**

Of the 1,066 investigated adults (mean age 35 ± 11.7 years; 49% females), 12.3% (95%CI 10.2;14.7) had periodontitis. Calcium intake had a direct protective effect on periodontitis (risk ratio (RR) 0.61; 95%CI 0.45;0.83), whereas no association between vitamin D and periodontitis was observed (RR 1.13; 95%CI 0.82;1.56). Stratified analyses revealed a null association between both vitamin D and calcium intake and periodontitis among men, but a protective association between calcium and intake and periodontitis among women (RR 0.56; 95%CI 0.38;0.79), while vitamin D remained without any association (RR 1.07; 95%CI 0.72;1.61).

**Conclusion:**

Our findings suggest a protective association between dietary calcium intake and periodontitis among women.

## 1 Introduction

Nutrition has been associated with a longer life expectancy and the prevention of several non-communicable systemic diseases, including type 2 diabetes and cardiovascular disease (1). In regards to oral health, the relationship between diet, especially rich in fermentable carbohydrates, and dental caries has been thoroughly investigated (2). Even though the association between nutrition and periodontitis, a chronic inflammatory disease affecting the supporting tissue of the teeth, has been explored in the literature, it seems this discussion can be further substantiated (3–6).

Although the local biofilm may influence the onset and progression of periodontal tissue destruction, environmental and genetic factors related to the host inflammatory response and their ability to solve it appear to account for approximately 80% of the periodontitis risk (7–9). As nutrition influences the ability of the immune system to mount and modulate inflammatory responses properly, it is possible to speculate a relationship between diet and periodontitis (1). While vitamin C seems to be the most investigated micronutrient possibly associated with periodontitis, considerable attention has also been given to the role of vitamin D and calcium (6). Nevertheless, the topic has not been fully clarified in the literature yet.

This interest relies on the potential mechanisms underlying the relationship between vitamin D, calcium, and periodontitis, which involve immune and hormonal effects that vary with sex. Vitamin D affects the inflammatory response by promoting macrophage shifting phenotype from their primary pro-inflammatory response (M1) toward an anti-inflammatory (M2) pattern (10). A similar effect of vitamin D is also observed among B- and T-cells, as the release of pro-inflammatory cytokines is inhibited, concomitant to an enhanced expression of anti-inflammatory cytokines (11). Additionally, calcium absorption is highly dependent on vitamin D levels. Low vitamin D levels reduce calcium absorption, which in turn upregulates parathyroid hormone release, osteoclastogenesis, and bone resorption to prevent hypocalcemia, thus, increasing bone loss (12). On a related note, low calcium intake has been associated with a greater risk of alveolar bone loss related with periodontitis (13). This mechanism, however, may differ from man to woman, as osteoclasts possess estrogen receptors but no androgen receptors (14). It is of utmost importance to explore the role of sex in this relationship.

Studies investigating the association between vitamin D and calcium intake with periodontitis have reached conflicting results. A systematic review found three observational studies exploring the association between dietary vitamin D intake and periodontitis, one of which indicated a null association, while the other two suggested a protective effect (6). Although more studies on the association between serum vitamin D levels and periodontitis are available, they yielded inconsistent results. Antonoglou et al. demonstrated that dietary vitamin D deficiency was associated with a higher prevalence of periodontitis (15), whereas Lee and colleagues found no association (16). Hence, to date, the role of vitamin D, if any, in periodontitis is still unclear. Similar findings were also observed in clinical trials. The available evidence about vitamin D supplementation (alone or combined with calcium), in part, revealed small effects among short-term studies (17–19).

On a similar note, the association between calcium intake and periodontitis is not yet evident. While some studies have indicated a detrimental effect of a calcium-deficient diet on periodontitis (20–22), others failed to identify any association (23). Despite the growing literature on the topic (5, 6, 24), few studies in periodontology have evaluated the dynamic association involving vitamin D and calcium intake. Thus far, most studies have focused on exploring the isolated effect of either vitamin D or calcium intake, neglecting their correlation (25). Either way, the use of conventional regression analysis fails to account for this complex relationship, and this might explain the controversial results found in the literature. In this case, an analytical approach that considers this framework may elucidate this matter further.

Thus, it becomes evident from the above the need for population-based studies with a large sample and the use of proper statistical methods to clarify the relationship between dietary vitamin D and calcium and their association with periodontitis. Accordingly, this study aimed to evaluate the direct and indirect relationship between dietary intake of vitamin D and calcium with periodontitis in adults from a population-based cohort study in Southern Brazil and assess whether these relationships differ between males and females.

## 2 Materials and methods

### 2.1 Participants and sampling procedures

**FIGURE 1:**
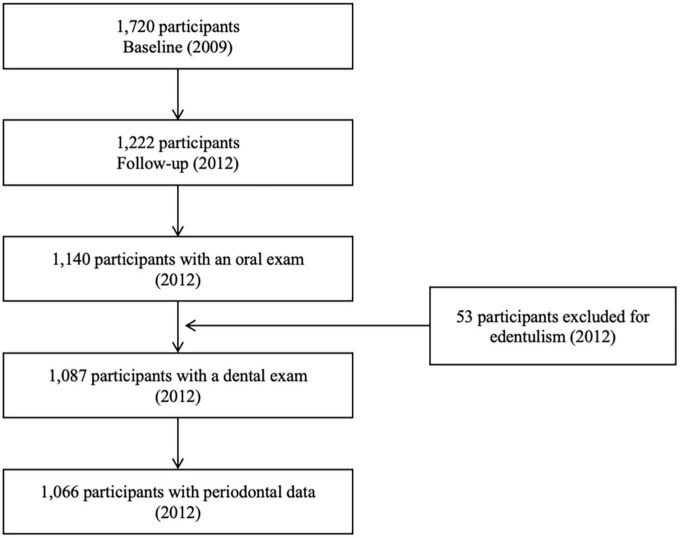
Flowchart of the EpiFloripa cohort study.

This study used data from the EpiFloripa Cohort Study, a population-based prospective study conducted in Florianópolis, a state capital in Southern Brazil. The baseline sample size (*n* = 1,720) was estimated considering the reference population between 20 and 59 years of age living in the urban area of the city in 2009. Sample size calculation accounted for a cluster sampling selection. Initially, 1/7 of all 420 census tracts of the city were selected in each household income decile; then, the households (1,134/16,755) were systematically chosen within the nominated census tracts. Adult residents in each house were considered eligible if aged between 20 and 59 years. Exclusion criteria comprised the presence of a severe physical or neurological impairment. In 2012, all participants were re-contacted for a follow-up examination (*n* = 1,066) (Figure 1). Further details on the study methodology (sampling and eligibility criteria) are available elsewhere (26).

The study was conducted in accordance with the Helsinki Declaration of 1975, as revised in 2013. The Ethics Committee in Human Research of the Federal University of Santa Catarina (351/2008 and 1772/2011), Brazil, approved the study. All participants signed a written consent form. The Strengthening the Reporting of Observational Studies in Epidemiology (STROBE) guidelines were followed to report the study.

### 2.2 Periodontitis: Outcome

In 2012, eight dentists performed dental examinations on all cohort members to assess for dental caries and periodontal outcomes at participants’ homes. Headlamps were used to improve visualization. In addition, dental examinations were standardized before fieldwork by comparing the results of the oral examinations performed on 20 adults (not cohort members) against a gold standard (27).

The periodontal examination of the cohort members included assessing the clinical attachment level (CAL) and bleeding on probing (BOP). Two diagonal quadrants were randomly selected according to the participant identification number. Six sites per tooth were examined using a ball-point periodontal probe (WHO probe) in the chosen quadrants. CAL was measured in mm and later dichotomized as absent (0-3 mm) or present ≥ 4 mm, while BOP after 15 s of probing was recorded as present. The outcome of the present study (periodontitis) was defined as the presence of CAL (≥ 4mm) and BOP in the same tooth (28).

### 2.3 Dietary vitamin D and calcium intake: Exposure

Information on dietary intake was elicited in 2012 using two 24h-dietary recalls. Data were collected following the “Multiple Pass” method (29), consisting of three stages: a “quick list,” a detailed description of food and beverage items consumed, and a review. After all cohort members completed the first 24 h-dietary recall, 40% of the participants were randomly selected for a second 24 h-dietary recall. The second recall was structured so that participants would report a week and a weekend day to capture the diversity of food consumed.

Dietary information gathered from the food recalls was entered into the Nutrition Data System for Research (NDSR) software from the University of Minnesota Nutrition Coordinating Center, USA, following the validation proposed by Fisberg and coworkers (30) for the Brazilian context. At this stage, all food sources were converted into grams, milliliters, or liters according to Brazilian standards. As the NDSR software uses information from the United States Department of Agriculture, typical Brazilian aliments not found in the software database had their nutritional values estimated and inserted in the NDSR software following Brazilian guidelines. Nutritional values related to total energy intake, vitamin D, and calcium were calculated for all participants. Subsequently, data from the two 24-hour recalls were used to adjust for the intra- and interindividual variability to reflect the usual intake. The Iowa State University (ISU) method was used for symmetrical food/nutrients variables without zeros in their distribution (TEI and TCVUPP), and the National Cancer Institute method (NCI) (31) was used for variables with a non-normal distribution and/or occasionally consumed nutrients. Both nutrient intake variables considered adjustment for the total energy intake – nutrient residual (energy-adjusted model) – recommended by Willet et al. (32). For analytical purposes, vitamin D and calcium intake were included as continuous variables in the model.

### 2.4 Covariates

Sex, age group (20/39 or 40/59 years), household income (in tertiles), smoking status (never, former, or current smoker), and waist circumference, an indicator of central obesity, (all collected in 2009) were considered potential confounders and included in the analytical models accordingly. While the former information was elicited from questionnaires, waist circumference (in cm) was measured in the narrower trunk region or at the midpoint between the last rib and the upper border of the iliac crest when the narrower trunk region was not apparent, using an inelastic tape measure (Sanny^®^, São Bernardo do Campo, São Paulo, Brazil) of 160 cm in length and a precision of 1 mm. Waist circumference was categorized into quartiles, and individuals in the last quartile were considered obese.

### 2.5 Theoretical framework

**FIGURE 2:**
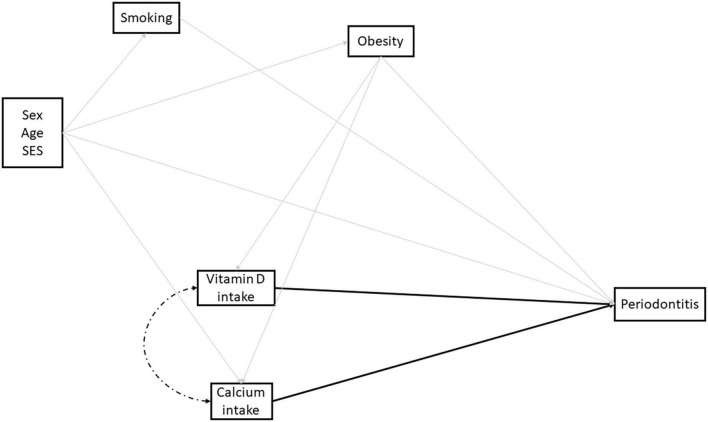
Directed acyclic graph depicting the relationship between dietary vitamin D and calcium and periodontitis among Brazilian adults. Solid light gray lines indicate potential confounders included in the analysis, whereas solid black lines indicate the direct effect of dietary intake of vitamin D and calcium on periodontitis. Finally, the dashed black line indicates the influence of vitamin D and calcium intake on each other.

Based on the literature, a directed acyclic graph was drawn to depict the relationship between micronutrients intake and periodontitis, given a set of potential confounders supported by previous evidence (Figure 2). We considered the intake of both micronutrients as potential exposures and established interaction between them, as indicated in the literature (25).

### 2.6 Analytical approach

Descriptive analysis of all variables used in the study is provided as absolute and relative frequencies for categorical variables and means with their respective standard deviations for continuous variables. Analyses were conducted using sampling weights clustered to the census sector, accounting for the inverse of the selection probability in 2009 and the probability of participating in 2012.

Given our complex analytical scenario comprising two highly correlated exposures, vitamin D and calcium intake, marginal structural modeling (MSM) appears as a valuable asset to performing multivariable analyses that model complex relations among a set of variables. It also allows the estimation of marginal risk ratios considering the counterfactual scenario. For this study, we calculated the controlled direct effect of vitamin D and calcium intake on periodontitis by estimating the inverse probability weight of both variables independently given the confounders and later multiplying them to obtain the final stabilized weight. In order to check the consistency of our final stabilized weight, a mean value of approximately 1.00 was expected.

Furthermore, we conducted a sensitivity analysis for unmeasured confounding by calculating the E-value, as proposed by VanderWeele and Ding (33). Briefly, a large E-value indicates that considerable unmeasured confounding would be required to eliminate the association between the exposures and outcome. All analyses were conducted using the Stata 16.1 (StataCorp., College Station, TX) software.

## 3 Results

Of the 1,222 participants evaluated in 2012, periodontal data were available for 1,066 adults (Table 1). At baseline, nearly 49% of the participants were females with a mean age of 35 years (SD ± 11.7), 58% were never-smokers, and 14% were classified as having central obesity. The total energy intake was 2,341.2 Kcal in males (SD ± 266.8) and 1,818.1 in females (SD ± 240.7), which was higher than the basal metabolic rate (1,676.1 Kcal SD ± 10.3 and 1,313.3 Kcal SD ± 7.1, respectively). The median difference between the total energy intake and the basal metabolic rate was 541 Kcal (interquartile range 381 – 726 Kcal).

**TABLE 1:** Sample characteristics and distribution according to periodontitis.

	*n* (%)^1^ or mean (SD)^2^	Periodontitis %^1^ or mean^2^ (95%CI)	
		No	Yes
**Sex^1^**			
Male	463 (49.3)	85.4 (81.8; 88.4)	14.6 (11.6; 18.2)
Female	603 (50.7)	90.0 (87.6; 92.0)	10.0 (8.0; 12.4)
**Age^1^**			
20-39	562 (63.9)	93.6 (90.7; 95.6)	6.4 (4.4; 9.3)
40-60	504 (36.1)	77.4 (72.3; 81.4)	22.6 (18.6; 27.1)
**Equalized income in Brazilian reais^**1**,**2**^**			
3rd tertile (highest income)	662.7 (270.4)	91.2 (87.0; 94.2)	8.8 (5.8; 13.0)
2nd tertile	1,585.7 (355.8)	87.6 (83.8; 90.6)	12.4 (9.4; 16.2)
1st tertile (lowest income)	4,775.5 (4,154.0)	83.8 (79.4; 87.4)	16.2 (12.6; 20.5)
**Smoking status^**1**^**			
Never-smoker	584 (58.8)	90.5 (87.6; 92.7)	9.5 (7.3; 12.4)
Former smoker	285 (23.9)	86.2 (81.4; 89.9)	13.8 (10.1; 18.6)
Current smoker	191 (17.3)	80.5 (75.3; 84.8)	19.5 (15.1; 24.7)
**Central obesity^**1**^**			
Eutrophic	866 (86.1)	89.2 (86.7; 91.4)	10.7 (8.6; 13.3)
Obese	169 (13.8)	76.7 (69.6; 82.3)	23.0 (17.0; 30.4)
**Vitamin D intake (μg)^**2**^**	4.4 (4.4; 4.5)	4.4 (4.4; 4.5)	4.5 (4.5; 4.6)
**Calcium intake (mg)^**2**^**	744.1 (729.0; 759.2)	748.7 (733.1; 764.2)	711.6 (692.3; 730.9)
**Periodontitis^**1**^**			
No	910 (87.7)	–	–
Yes	156 (12.3)	–	–

^1^n (%); ^2^Mean (SD).

The overall prevalence of periodontitis was 12.3% (95%CI 10.2;14.7), and the average vitamin D and calcium intake were 4.4 μg (SD ± 0.4) and 744.1 mg (SD ± 109.1), respectively. Correlations between vitamin D and calcium intake were 0.31 for the whole sample, 0.42 among men, and 0.30 among females. While vitamin D intake was similar among participants periodontally healthy (4.4 μg) or with periodontitis (4.5 μg), the latter had lower levels of calcium intake (748.7 and 711.6 mg, respectively). A higher prevalence of periodontitis was also observed among males, older adults, current smokers, centrally obese, and the poorest (lowest income tertile). Regarding sex, men had an average intake of 4.5 μg of vitamin D and 714.9 of calcium, while women had 4.3 μg and 773.2, respectively.

Table 2 displays the risk ratios (RR) with their respective 95% confidence intervals (95% CI) for the total sample and separately for men and women. Calcium intake protected from periodontitis in the whole sample (RR 0.61; 0.45;0.83), whereas no association between vitamin D and periodontitis was observed (RR 1.13; 95%CI 0.82;1.56). The same null association between vitamin D intake and periodontitis was observed among males (RR 1.13; 95%CI 0.65;1.97) and females (RR 1.07; 0.72;1.61). However, there was evidence of heterogeneity in the association between calcium intake and periodontitis (*P*-value for interaction 0.018), as the protective association between calcium intake and periodontitis was observed in women (RR 0.55; 95%CI 0.38;0.79) but not among men (RR 0.69; 95%CI 0.40;1.14).

**TABLE 2:** Association between vitamin D, calcium, and periodontitis estimated using marginal structural modeling.

	Total sample	Men	Women
	
	Risk ratio (95%CI)	Risk ratio (95%CI)	Risk ratio (95%CI)
Vitamin D intake	1.13 (0.82; 1.56)	1.13 (0.65; 1.97)	1.07 (0.72; 1.61)
Calcium intake	0.61 (0.45; 0.83)	0.69 (0.40; 1.14)	0.55 (0.38; 0.79)
*E*-value for calcium intake^1^	2.66 (1.70)	–	3.04 (1.84)

Estimates given are risk ratios and respective 95% confidence intervals. Analyses are shown for the total sample, as well as stratified by sex.

^1^The *E*-value was calculated only for positive associations. Thus, there is no values for vitamin D intake. The *E*-value provides the point estimate and lower limit of the confidence interval (between brackets).

Finally, our sensitivity analysis for unmeasured confounding shows that the strength required for an unmeasured con-founder to eliminate the effect of calcium intake on periodontitis would be 3.0 (Table 2).

## 4 Discussion

Our findings suggest that a high vitamin D intake was not associated with periodontitis, while high dietary calcium intake had a protective, though weak, effect on periodontitis, especially in women. These results could be noticed by using an analytical approach able to deal with the complex relationship between vitamin D and calcium intake and periodontitis. Thus, it is possible to speculate that the conflicting results found in the literature regarding this association could be partially attributed to analytical methods used thus far, as conventional regressions do not support exploring intertwined exposure variables, as in our study.

Prior to discussing our findings, we should carefully examine the limitations inherent to our study. Firstly, although we used data from a cohort study, our analysis is cross-sectional, as information on dietary intake and periodontitis was elicited in 2012. However, we do not believe that periodontitis might have impacted calcium and vitamin D intake, as even severe cases of periodontitis would not prevent individuals from eating foods rich in these nutrients. Thus, the chance of reverse causality, if any, is trivial. Secondly, our periodontal data did not allow the employment of the classification system recommended by the two major periodontal organizations, i.e., the American Academy of Periodontology and the European Federation of Periodontology, nor an approach that would account for the multidimensional aspect of periodontitis (latent). While this might have underestimated our results, other studies using a similar classification, including the Global Burden of Diseases, observed a prevalence analogous to ours (34). Moreover, we found comparable results from other classification systems when examining the association between periodontitis and obesity using the current periodontal classification (28).

Additionally, the intake of vitamin D and calcium was assessed using a 24h-dietary recall. It is possible to speculate that eliciting this information only twice (once in the total sample and replicated in a random subsample) might not have captured the habitual food consumption variation. However, replication rates of >40%, as in our case, may not lead to a loss of dietary assessment precision (35). In addition, one might speculate about the possibility of underreporting the total energy intake by the participants, which was identified in only 2.3% of the cohort. Thus, it is unlikely that underreporting has affected our results, as the use of the multiple pass method to assist participants in remembering their food intake aimed to minimize this source of bias.

Furthermore, although one might assume that vitamin D and calcium supplementation might have biased our results, in 2009, only 2.4% of our sample had used supplements containing vitamin D and calcium (data not shown), hence, not impacting our findings. Moreover, while calcium levels highly depend on vitamin D, we did not assess calcium and vitamin D serum levels. In the EpiFloripa study, for instance, a correlation of 0.06 was observed between intake (assessed in 2012) and serum levels of vitamin D (collected in 2014) (data not shown). Given the weak, if any, correlation between vitamin D intake and serum levels [25(OH)D] (36), it is possible to speculate that the null association between vitamin D and periodontitis might have resulted from the lack of data on 25(OH)D. Another potential explanation for this null association might relate to the age of our sample, as an inverse association between vitamin D and periodontitis was observed only among Americans over 50 years (37). Therefore, future studies with a broader age sample are encouraged to clarify this relationship.

It is also relevant to mention that few individuals in this study had a vitamin D and calcium intake considered appropriate following the current recommendations (38). Although the prevalence of inadequate intake of calcium and vitamin D is high in Brazil, calcium and vitamin D intake in our cohort is higher than the national as well as the regional average (Southern Brazil) (39). Finally, we did not have data on sun exposure, an important factor influencing the serum levels of vitamin D. Still, a recent study also conducted in Southern Brazil, in a geographical region where sun exposure is similar to ours, indicated a positive effect of sun exposure on serum levels of vitamin D, among men, but not among women, whose crucial factor was vitamin D intake (40). Therefore, it is possible to speculate that our results would not have been extensively impacted by adding this information to our analytical models, but future studies should investigate this topic. On a similar note, our sensitivity analysis for unmeasured confounding revealed that only variables with an approximately 3-fold association with the outcome would eliminate our results, hereafter supporting the robustness of our findings.

The nutritional assessment and analysis and the representativeness of the sample concerning the target population are among the strengths of our study. Furthermore, the thoughtful analytical approach should be pointed out. Most, if not all, studies investigating the relationship between vitamin D, calcium, and periodontitis did not account for the complex dynamic between these variables by neglecting the relationship between vitamin D and calcium. This might explain the conflicting results regarding this relationship in the literature, as evident in the systematic reviews conducted on the topic, where the results vary from negative to null and positive (4–6).

Other factors that may explain the lack of consistent results regarding the association between vitamin D, calcium, and periodontitis relate to the assessment of the micronutrients. In a study using Mendelian randomization, the authors did not find an association between 25(OH)D serum levels and periodontitis in a sample of approximately 45,000 Europeans (41). While that study corroborates our findings regarding the lack of association between vitamin D intake and periodontitis, the authors did not use the information on calcium. The potential lack of direct association between vitamin D intake and periodontitis may be explained by studies using calcium tracers. Even though appropriate 25(OH)D levels increase the calcium absorption efficiency, a serum concentration of at least 50 nmol/L is necessary (42). Therefore, vitamin D supplementation does not enhance calcium absorption efficiency in most healthy humans. However, future studies should focus on the interplay among the serum levels of vitamin D, calcium, parathyroid hormone, metabolic dysfunction, and periodontitis.

From a biological perspective, a protective, though weak, association between calcium but not vitamin D and periodontitis was found in women. However, low vitamin D levels reduce calcium absorption, upregulating parathyroid hormone release, osteoclastogenesis, and bone resorption to prevent hypocalcemia (12). Systematic reviews (43, 44) have not identified a significant impact of vitamin D supplementation alone on the risk of fractures, whereas calcium supplementation, mainly in combination with vitamin D, had a protective effect on fractures. Thus, it is possible to speculate a similar pattern in periodontitis. While a positive effect of vitamin D and calcium on bone mineral density is noticed, it appears that this combination also normalizes Parathyroid Hormone Intact (PTHi) and 25(OH)D levels, which, in turn, regulate serum calcium concentration and bone remodeling, especially among women (45). Moreover, studies argue that women tend to store more fat than men and that fat tissue sequester vitamin D from serum (46). Reduction in vitamin D levels upregulates intracellular calcium accumulation in adipocytes, resulting in lipogenesis and weight gain (47). In addition, as osteoclasts possess estrogen receptors but no androgen receptors (14), factors influencing the bone remodeling process become more evident among women, thus, explaining our findings.

## 5 Conclusion

Our findings suggest a small protective association between dietary calcium intake and periodontitis among women. However, further studies accounting for the dynamic relationship between vitamin D and calcium (intake and serum levels) are needed to further elucidate this association.

## Data availability statement

The original contributions presented in this study are included in the article/supplementary material, further inquiries can be directed to the corresponding author/s.

## Ethics statement

The studies involving human participants were reviewed and approved by the Ethics Committee in Human Research of the Federal University of Santa Catarina (351/2008 and 1772/2011). The patients/participants provided their written informed consent to participate in this study.

## Author contributions

GN contributed to conception, data analysis and interpretation, and drafted and critically revised the manuscript. FL contributed to the data analysis and interpretation and critically revised the manuscript. DG-C and KP contributed to the conception and design and critically revised the manuscript. MP contributed to the conception and design, data interpretation, and critically revised the manuscript. All authors gave final approval and agreed to be accountable for all aspects of the work.
